# Vascular hypothesis of Alzheimer’s disease: role of apoE and apoE receptors

**DOI:** 10.1186/1750-1326-8-S1-O20

**Published:** 2013-09-13

**Authors:** Guojun Bu, Chia-Chen Liu, Takahisa Kanekiyo

**Affiliations:** 1 Department of Neuroscience, Mayo Clinic, Jacksonville, Florida 32224, USA

## Background

Alzheimer’s disease (AD) is a progressive neurodegenerative disease that has emerged as the most prevalent form of late-life dementia in humans [1]. Production of amyloid-β (Aβ) from the amyloid precursor protein (APP) and its subsequent accumulation, aggregation and deposition in the brain are central events in the pathogenesis of AD [1]. Cerebral amyloid angiopathy (CAA) is a major pathological feature of AD where amyloid spreads and deposits throughout the blood vessel walls in the central nervous system. These pathogenic events induce a specific clinical presentation profile including cerebral hemorrhage, stroke, ischemic infarctions, subarachnoid hemorrhage, seizures, cognitive impairment and dementia [2]. While Aβ is a key molecule in AD, epidemiological studies have shown that several well-established risk factors for AD, including diabetes mellitus, atherosclerosis, stroke, hypertension, transient ischemic attacks, microvessel pathology and smoking, have a vascular component that reduces cerebral perfusion [3]. In fact, detection of regional cerebral hypoperfusion through neuroimaging techniques can preclinically identify individuals at risk for AD. Further, cerebral hypoperfusion precedes hypometabolism, cognitive decline, and neurodegeneration in AD [3]. Therefore, disturbance of cerebrovascular system is likely a major contributor to AD pathogenesis. Among the three human apolipoprotein E (apoE) isoforms (E2, E3 and E4), *APOE4* is the strongest genetic risk factor for late-onset AD. The most consistent finding that differentiates apoE4 from apoE3 is their respective roles in brain Aβ clearance, where apoE4 is less efficient than apoE3 in promoting Aβ clearance [4]. In addition, *APOE4* also increases the risk for CAA and vascular dementia [4]. Because apoE4 is known to damage blood-brain barrier (BBB) integrity and reduces small cerebral vessels [5], apoE is likely involved in the maintenance of cognitive function through regulating the function of cerebrovascular systems.

## Materials and methods

We generated conditional knockout mice deleting a major apoE and Aβ receptor LRP1 in vascular mural cells (smLRP1-KO), which include smooth muscle cells and pericytes. These mice were further bred to the background of amyloid model mice APP/ PS1. Cerebral blood flow, behaviors and Aβ metabolism were compared among human *APOE* isoform (E2, E3 and E4)-targeted replacement (TR) mice and between smLRP1-KO mice and wild-type littermate controls at young and old ages.

## Results

We found that cerebral blood flow and memory performance are reduced, while endogenous Aβ levels are elevated, in aged *APOE4-*TR mice compared to *APOE3-*TR mice and in smLRP1-KO mice compared to controls. When crossed with APP/PS1 mice, deletion of LRP1 in vascular mural cells exacerbated Aβ deposition both in the cortical parenchyma as amyloid plaques and along the cerebral vessels as CAA [6].

## Conclusions

Our results demonstrate that the presence of APOE4 or an absence of apoE receptor LRP1 leads to cerebrovascular defects, which compromise Aβ clearance machinery resulting in Aβ accumulation in the brain. The resulting Aβ aggregation and deposition further exacerbate cerebrovascular dysfunction in AD.
